# Experimentally Guided Computational Model Discovers Important Elements for Social Behavior in Myxobacteria

**DOI:** 10.1371/journal.pone.0022169

**Published:** 2011-07-19

**Authors:** Melisa Hendrata, Zhe Yang, Renate Lux, Wenyuan Shi

**Affiliations:** 1 Department of Mathematics, California State University Los Angeles, Los Angeles, California, United States of America; 2 Molecular Biology Institute, University of California Los Angeles, Los Angeles, California, United States of America; 3 School of Dentistry, University of California Los Angeles, Los Angeles, California, United States of America; University of Vermont, United States of America

## Abstract

Identifying essential factors in cellular interactions and organized movement of cells is important in predicting behavioral phenotypes exhibited by many bacterial cells. We chose to study *Myxococcus xanthus*, a soil bacterium whose individual cell behavior changes while in groups, leading to spontaneous formation of aggregation center during the early stage of fruiting body development. In this paper, we develop a cell-based computational model that solely relies on experimentally determined parameters to investigate minimal elements required to produce the observed social behaviors in *M. xanthus*. The model verifies previously known essential parameters and identifies one novel parameter, the active turning, which we define as the ability and tendency of a cell to turn to a certain angle without the presence of any obvious external factors. The simulation is able to produce both gliding pattern and spontaneous aggregation center formation as observed in experiments. The model is tested against several known *M. xanthus* mutants and our modification of parameter values relevant for the individual mutants produces good phenotypic agreements. This outcome indicates the strong predictive potential of our model for the social behaviors of uncharacterized mutants and their expected phenotypes during development.

## Introduction

Organized movement of cells is an important event in a variety of biological processes. In multicellular organisms such as vertebrates, organogenesis and morphogenesis require organized migration and passing of signals between cells [1]. As an example for unicellular eukaryotes, the slime mold *Dictyostelium discoideum* achieves highly organized cell movement in its different pattern formation by relaying diffusible morphogens [2], [3]. As representatives for prokaryotes, the myxobacteria display organized gliding patterns during vegetative swarming and form fruiting bodies with various shapes during development, demonstrating their versatility of organized cell movement [4]. While the life cycle and social behaviors of myxobacteria resemble in many respects those of cellular slime molds, the mechanisms to achieve these behaviors differ. Directed motility in *Dictyostelium discoideum* is based on chemotaxis where cells sense and respond to chemoattractant gradients, resulting in a long-range cell interactions [2]. In contrast, myxobacteria rely on direct local contact dependent signaling and social interactions between neighboring cells to coordinate cell movement [4]. In order to clearly delineate the cellular interactions and identify essential components required for organized movement, *Myxococcus xanthus* is frequently chosen as a bacterial model system.

*Myxococcus xanthus* is a gram-negative bacterium initially isolated from cultivated soil. Individual *M. xanthus* cells are elongated, rod-shaped, about 3–5 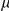
m in length and 0.5 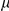
m in width. They do not have flagella and are therefore unable to swim. Instead, the cells glide on solid surfaces using two distinct motility systems: Adventurous (A)-motility and Social (S)-motility [5]. Single cell movement via A-motility is the preferred type of locomotion on dry surfaces, while coordinated movement via S-motility is mainly utilized on moist surfaces, enabling the bacterium to adapt to a variety of physiological and ecological environments [6]. Type IV pili (TFP), the molecular motors for S-motility, are found at the leading pole of the cells. They function by extending the pili at one cell pole, attaching to surfaces or to another cell and then retract, thereby pulling the cell forward [7]–[9]. The cell surface extracellular polysaccharide (EPS) was found to be the anchoring substrate for TFP and trigger retraction [10]. The A-motility engine, on the other hand, is initially thought to be localized at the lagging pole of the cell, powered by the secretion of a gel-like slime through nozzle-like structures, and generate a propulsive force to push the cell forward [11], [12]. Although the chemical composition of the slime is not yet determined, it is suggested to include repeat unit polysaccharides [12]. Alternatively, a focal adhesion model is proposed to explain A-motility [13], [14]. In this model, transient adhesion complexes push against the surface and gain traction with the aid of extracellular polysaccharide slime, which enables the cells to move forward in a rotating manner [15]. Although the existing models do not agree regarding the nature of the A-motility engine, both support the excretion of EPS slime on surfaces. In addition, motile *M. xanthus* cells frequently reverse their gliding directions at 6 to 8 minute intervals [16] by changing the use of the two motility systems between opposite cell poles. The synchronization of the two motors is obtained by spatial oscillations of the corresponding motility proteins [17].

Individual bacterial cell behavior changes in groups and during the complex life cycle of *M. xanthus*, resulting in the most distinct feature of *M. xanthus* - its social phenotype. During vegetative growth, *M. xanthus* cells use their two motility systems to glide across surfaces of soil particles, or on agar surfaces in the laboratory. During colony formation the cells locally align into domains [18]. Under these conditions, cells glide away from the center of a colony towards an area where they retrieve new nutrients from prey that are lysed by their secreted autocides [19]. When nutrients are depleted, *M. xanthus* cells change their gliding direction from outward to inward and eventually form multicellular dome-like structures called fruiting bodies. During this process, cells stop growing and merge into streams that then join to form initial aggregation centers. It is proposed that the initial aggregate nucleus or kernel may result from a random traffic jam which is later resolved [20]. Cells in the early aggregation centers are motile and large spiral patterns are formed in monolayers on the substratum [21]. These orbiting patterns may persist into later stages of development at the bottom of fruiting bodies [22]. Small adjacent aggregation centers fuse to form larger mounds. When more cells are absorbed into the mounds, they rise up and increase in size and eventually form fruiting bodies. Cells within the fruiting body develop into metabolically dormant myxospores and these myxospores will germinate and become vegetative again when nutrients become available [4].

For a long time, continuing attempts have been made to simulate the development of *M. xanthus* focusing on different stages [23]–[30]. However, some of these models are incomplete in capturing important biological properties (e.g. cell reversal and quorum sensing are excluded), while others are overwhelmed by inaccuracy due to the implementation of artificial parameters that were not experimentally determined. In this study, we develop a cell-based model that takes only experimentally determined parameters into account for identification of the minimal elements required to produce the observed gliding patterns and aggregation center formation during the early stage of *M. xanthus* development (up to 12 hours). Our model verifies the known essential parameters for early aggregation center formation in *M. xanthus* development, which is the key event in fruiting body formation. Furthermore, we identify one novel parameter, the *active turning*, which is defined as the action of a cell to turn its cell body. This results in changing the direction of movement at a certain angle without the presence of any obvious external factors. Our simulation demonstrates that this active turning parameter is in fact essential in producing the observed gliding pattern and in facilitating efficient and spontaneous aggregation center formation. We further test our model against the social phenotypes of several known *M. xanthus* mutants and good agreements with experimental observations are obtained. This suggests that the model can be used as an effective tool in predicting the phenotypes of mutants with defects in any of the parameters important for social behaviors of *M. xanthus*.

## Results

To understand cell behavior, we utilize individual cell motility analysis (Materials and Methods) and experimentally observe the following elements that were previously described to be essential for *M. xanthus* gliding and aggregation center formation during early development.

### 1. Basic cellular properties

*M. xanthus* are elongated, rod-like cells gliding on surfaces at an average rate of about one cell length per minute. Cells often align parallel with each other when they move in close proximity or collide. This alignment is achieved by type IV pili attachment to the EPS of a neighboring cell and pulling the cells together. However, the cells moving side-by-side do not always adhere, they sometimes depart.

### 2. Cellular reversal

*M. xanthus* cells change direction not by making U-turns, but by reversing the cell's polarity [16]. The initial leading cell pole becomes the lagging pole, and vice versa. When isolated bacteria glide over the surface for a few body lengths, they pause shortly, reverse direction and move back on their original path, at intervals of about 6–8 minutes. The frizzy (Frz) chemosensory system regulates these reversal events [16].

### 3. Quorum sensing

Quorum sensing is a cell-cell communication process in which bacteria use the production and detection of extracellular chemicals called autoinducers to monitor the cell population density. Quorum sensing allows bacteria to switch between different gene expression programs: one favored at low-cell-density and another favored at high-cell-density [31]. In *M. xanthus*, cells can sense cell density and respond accordingly. One inducer is proposed to be the 17-kDa cell surface protein called C-signal, which accumulates by contacts between cells in high cell density and rippling events [32]–[34]. C-signal carries information regarding cell density and cell position with respect to other cells [35]. During development, increased C-signal molecules interact with the Frz chemosensory system via a cascade of covalent modifications [36]. Significant increase in the methylation of FrzCD [37] reduces the cell reversal frequency. EPS, which is also found to accumulate to a high concentration during development, may also be a quorum sensing inducer [26]. Experimental data on the correlation of reversal frequencies with different cell densities was previously reported by Shi et al. [38] and is similar to findings by Jelsbak et al. [34].

### 4. EPS slime production and following

When a *M. xanthus* cell glides over the surface, it leaves a slime trail that is evident as a bright line in phase contrast microscopy. This slime trail is suggested to contain a gel-like slime mainly made up of polysaccharides. The EPS portion of the extracellular matrix important for S-motility are also detected in the slime trails. EPS is important for guiding cell movement and for building up the scaffold that holds fruiting bodies together [39]. However, the source and actual components of EPS slime are not determined yet. In this paper, we use the general term “EPS slime” to refer to the material deposited by the cell on the surface. It has been shown that myxobacterial cells tend to follow a pre-existing EPS slime trail [40]. When a cell begins to cross an existing trail, it turns to an acute (less than 90 degrees) angle at the intersection to follow the trail. Cells are also observed to move in both directions on the same trail [4].

Employing the four parameters described above, we develop an off-lattice computational model to simulate myxobacteria gliding behavior and the pattern produced during the initial stage of development. This model is a cell-based model in which cell movement is governed entirely by a set of rules that reflect the properties of motility engines and local cell interactions. In this model, a cell is represented by a string of nodes, which are connected to one another by a segment of equal length. The use of multiple nodes allows the model to closely capture the cells' ability to turn and bend, and hence, geometric constraint and artificiality are minimized. The first node, which represents the cell's leading pole, determines the direction of movement and leads forward the rest of the body nodes. Each cell in our model is assigned a reversal clock that functions as a periodic timer to keep track of the cell's reversal period and adjust it according to the quorum sensing mechanism. A detailed description of the model and its motion algorithms are discussed in the Materials and Methods section.

**Figure 1:**
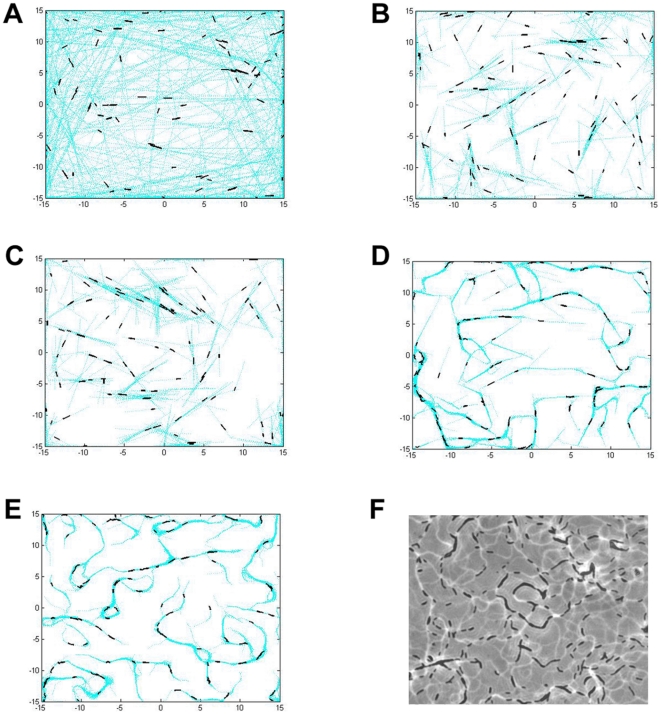
Simulation images of cells' gliding behavior showing the effect of various parameters to the gliding patterns. The following parameters listed in Table 1 are included in the model: (A) Basic cellular properties. (B) Basic cellular properties and polarity reversal. (C) Basic cellular properties, polarity reversal and quorum sensing. (D) Basic cellular properties, polarity reversal, quorum sensing and EPS slime production and following. (E) Basic cellular properties, polarity reversal, quorum sensing, EPS slime production and following, and active turning. (F) Experimental image of wild-type cells and their gliding pattern. All simulations start with 100 cells in a domain with periodic boundary conditions and run for 2 hours (360 simulation time steps).

Four different sets of simulations were carried out for various purposes. The first set of simulations (Fig. 1) was performed to test the necessity of the four parameters mentioned above in simulating myxobacteria gliding behavior prior to aggregation during development. The values for these parameters are summarized in Table 1, items 1–4. We start with only the basic cell parameters (Table 1, item 1) and sequentially include additional parameters to the model until all four are used. We simulate 100 cells, initially placed randomly over the surface with periodic boundary conditions, for two hours. EPS slime traces are plotted and shown as light blue dots.

**Table 1:** Experimentally determined parameters used in the model for simulating the gliding behavior and early aggregate formation during *M. xanthus* development.

	Parameters	Parameter description and values
1	Basic cellular properties	a. Cell length = 3–5 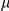 m. Length∶width ratio = 10∶1.
		b. Cell speed = 4.5 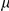 m/min [48].
		c. Physical interactions: two cells align with each other during pole-to-side collision; cell either slightly changes its orientation to pass by the colliding cell and move forward, or reverses during pole-to-pole collision.
2	Cellular reversal	Cells reverse from time to time. Initial reversal interval is set according to a random normal distribution with mean = 6.24 minutes, standard deviation = 0.5 minutes [42].
3	Quorum sensing	Cell density dependent reversal frequency modulation is determined by the number of neighboring cells as follows [38]:
		a. 8–12 minutes (5–19 neighboring cells)
		b. 15–30 minutes (20–99 neighboring cells)
		c. 30–40 minutes (100–999 neighboring cells)
		d. 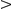 300 minutes ( 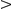 1000 neighboring cells)
4	EPS slime production and following	a. EPS slime production: As cell moves, it deposits EPS slime on the surface.
		b. EPS slime sensing: Cells detect EPS slime within one Type-IV pilus distance, which is approximately equal to one cell length [49].
		c. EPS slime following: Cell follows an existing EPS slime trail ahead of it to the direction with higher EPS slime concentration.
5	Active turning	a. Active turning angle: random normal distribution with mean = 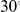 , standard deviation = 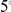 , maximum = 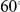 .
		b. Active turning frequency: normal distribution with mean = 3 minutes, standard deviation = 1 minute.

The basic cell parameters coupled with the cell motility algorithm described in the Materials and Methods section allow cells to move on the surface individually. In the absence of collision, the cell moves forward in the direction of their long axis leaving straight traces (Fig. 1A). In Fig. 1B, cellular reversal is incorporated into the model and cells are now able to reverse periodically with a fixed reversal frequency. As a result, they are no longer able to travel as far as in Fig. 1A. Their straight paths tend to be shorter and in absence of collision, cells simply glide back and forth following the same path. In Fig. 1C, reversal frequency is modulated according to the local cell density given in Table 1 item 3 - quorum sensing. However, at the cell density used in all simulations in Fig. 1, the quorum sensing does not appear to have a major impact on the cellular behavior. The chances in which cells will group together and alter their reversal frequency are low, and therefore the simulation result in Fig. 1C looks very similar to Fig. 1B. In the simulation shown in Fig. 1D, each cell is capable of depositing EPS slime while gliding over the surface and at the same time, sensing and following the EPS slime trail laid previously on the surface. The tendency of a cell to follow an EPS slime trail causes it to turn and change direction of movement more frequently. However, a number of long straight paths are still obvious, which is not commonly observed experimentally (Fig. 1F). This suggests that the four parameters are insufficient to accurately represent *M. xanthus* gliding behavior.

**Figure 2:**
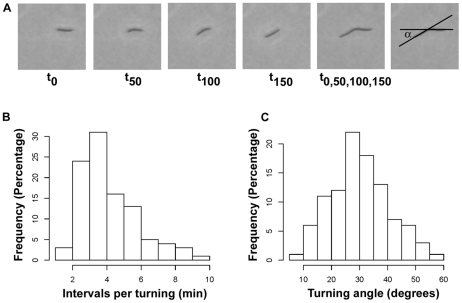
The active turning event in *M. xanthus*. (A) Active turning is commonly observed in individual *M. xanthus* cells. A series of snapshots from a recorded experiment taken at time 0 second, 50 seconds, 100 seconds, 150 seconds and their overlap image shows how active turning angle 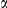 is determined. (B) The experimental value of the active turning frequency is normally distributed around 3 minutes per turn. (C) The experimental value of the active turning angle follows a normal distribution with a mean of approximately 30 degrees.

In order to improve the simulation and enable our model to fully represent *M. xanthus* gliding behaviors during development, we have identified one novel parameter that was overlooked before, the *active turning*. When single cells initially glide over agar surface, their movement paths do not always follow a straight line. In contrast, single cells are frequently found to change direction of movement without the presence of apparent external factors covered in the known parameters: collision, alignment with other cells, or following an existing EPS slime trail. We define this new cellular behavior as active turning. The active turning angle is measured as the angle between the cell long axis before and after one cell length movement (Fig. 2A). By examining the active turning events in hundreds of individual cells in recorded experiments, we find that the frequency of active turning is normally distributed with an average of approximately 3 minutes per turn (Fig. 2B). In addition, the turning angle is also normally distributed centered around 30 degrees and there is an equal chance for cells to either turn to the left or turn to the right (Fig. 2C).

Since active turning of a single cell is a common event observed during the initial stage of development, we hypothesize that this behavior is important for the early gliding pattern formation. We add the active turning property as the fifth parameter in our computational model using experimentally determined values (Fig. 2; Table 1 item 5). This addition greatly improves our previous simulation results. In particular, more circular EPS slime trails are now produced, as shown in Fig. 1E. These circular paths later become the starting locations for initial aggregation as more cells join and follow these paths. This result demonstrates that all five experimentally determined parameters are important for gliding pattern formation during *M. xanthus* early development.

**Figure 3:**
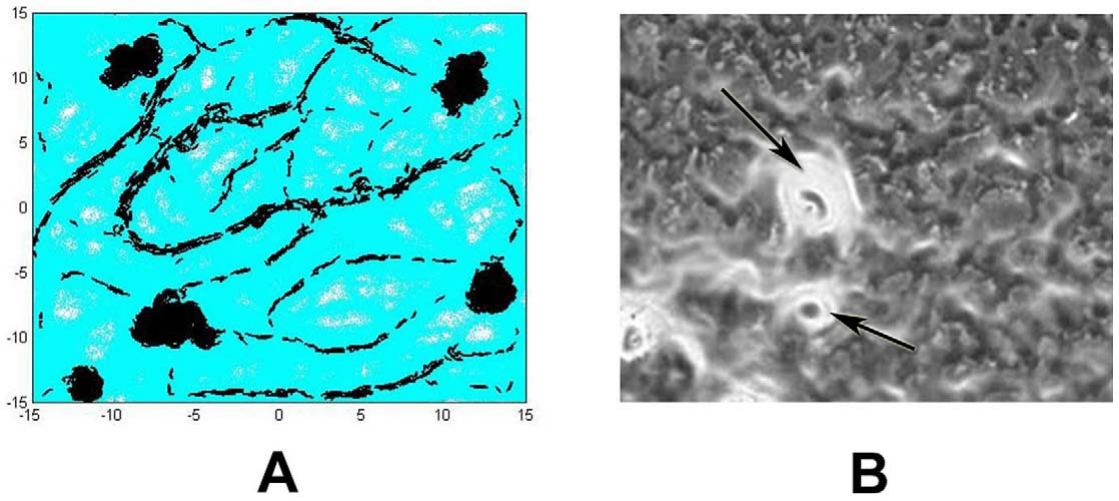
Aggregation center formation of wild-type cells at the end of 12 hours under developmental conditions. (A) Simulation image using all five parameters with values listed in Table 1. Simulation uses periodic boundary conditions and starts with 5000 cells for 12 hours (2160 simulation time steps). (B) Experimental image showing early aggregation centers at 12 hours after initiation of starvation. Cells are shown in dark grey and EPS in white. Arrows show aggregation centers formed by large number of cells with high concentration of EPS. At locations with high EPS concentration, bright white color overrides black in the view of microscope.

In the next simulation (Fig. 3A), we start from a higher cell density and test whether the five cellular properties are sufficient to produce initial aggregation centers similar to those observed in *M. xanthus* development assay (Fig. 3B). We simulate 5000 *M. xanthus* cells, with all five parameters that are set according to values listed in Table 1. The cells are initially placed randomly over the simulation domain with periodic boundary conditions, where they start to glide independently of each other. During gliding, they deposit EPS slime and follow existing slime trails. At this early stage, cells reverse their polarity periodically and their frequent turns are due to either collision between cells, EPS slime following, or the active turning itself. As several cells follow the same trail, they form small groups and move together in streams. Individual cells often join the streams, which results in increasing local cell density inside the streams. Due to the quorum sensing mechanism, cells that locate at a higher neighboring density modulate their reversal frequency. Streaming cells do not reverse as frequently as before and thus travel farther. As local cell density keeps increasing, cells in streams begin to turn to one direction once they encounter an area with high EPS slime concentration. This one directional turning event results in the formation of a circular path. With many cells following this path, it gradually forms an initial aggregation center in which cells orbit in spiral patterns. More cells are absorbed to this center causing it to become more condensed. At this stage, few cells leave the center occasionally and some may reverse to rejoin the center. Since we only simulate a fixed, limited number of cells approximately 90% of cells join the aggregation center at the end of 12 hours, leaving few cells gliding independently outside. This new model exactly reproduces different stages and the multicellular behavior during the aggregation center formation of *M. xanthus* development. Therefore, we demonstrate that the five parameters are not only necessary for creating the gliding pattern at low cell density but are essential in early aggregation center formation during *M. xanthus* development at higher cell density.

**Figure 4:**
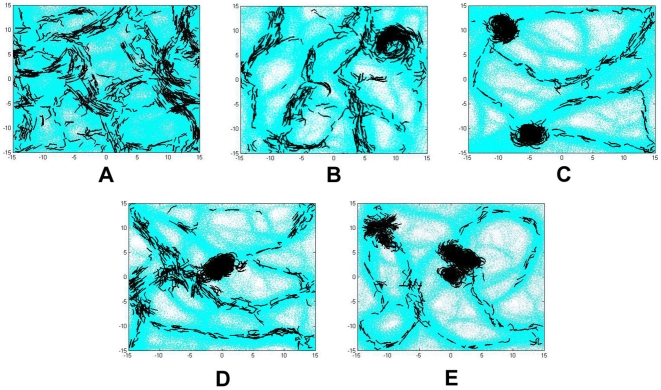
Simulation images of wild-type cells with various mean active turning angles at 12 hours of development. The active turning angle in these simulations are set according to random normal distribution with mean (A) 5 degrees, (B) 15 degrees, (C) 30 degrees, (D) 45 degrees, and (E) 60 degrees. Each simulation uses periodic boundary.

In the next simulation (Fig. 4) we aim to verify whether the experimentally observed 30 degree average turning angle is necessary for efficient aggregation. We repeat the simulation in Fig. 3A with several average turning angle distributions between 5 and 60 degrees for up to 20 hours and measure the time needed to complete the initial aggregation. Changing this parameter to values other than 30 degrees produces non-optimal results. The results are summarized in Table 3. An average active turning angle smaller than 30 degrees does not initiate any discernible aggregation process (5 degree angle) or produces incomplete structures (15 degree angle) within the 20 hour simulation period. Active turning angles with normal distributions that are centered at values larger than 30 degrees do not reflect the experimentally observed behavior even though they still allow the formation of aggregation center. In the case of 45 degrees, round aggregates are formed within approximately 9 hours. However, some of them are unstable and may break apart. In the case of 60 degrees, many small round aggregates clump together in regions with high EPS concentration. These small aggregates are unlikely to merge. Consequently, long aggregates with many centers are formed. Figure 4 shows simulation results of wild-type cells with different means of active turning angle distributions. These results indicate that an active turning angle, with a normal distribution centered around 30 degrees, together with the four other previously determined factors are essential for modeling spontaneous and efficient aggregation center formation during *M. xanthus* development.

**Figure 5:**
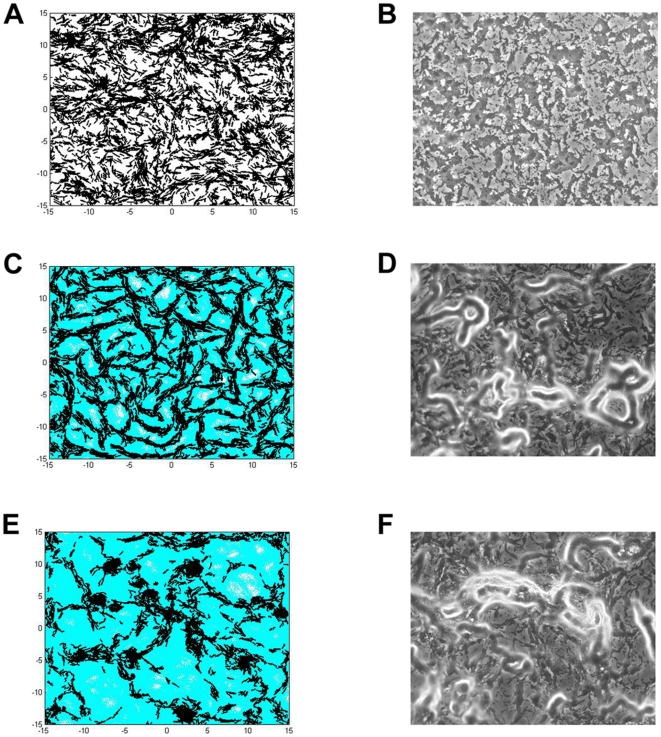
Simulation and experimental images of *M. xanthus* genetic mutants at the end of 12 hours during development. Panels A, C and E show simulation images with periodic boundary conditions for SW504 (no EPS), SW601 (elevated reversal frequency) and SW600 (reduced reversal frequency) mutants, respectively. All three simulations use periodic boundary conditions and run for 2160 time steps. Panels B, D and F show their corresponding experimental images. In these images, cells are shown in dark grey and EPS in white. At locations with high EPS concentration, bright white color overrides black in the view of microscope.

Using the five experimentally determined parameters, our computational model accurately simulates the early aggregation center formation during *M. xanthus* development. To further test this model, we simulate 5000 cells of several known genetic mutants during the first 12 hours of development, and test whether the simulation produces phenotypes similar to those observed experimentally (Fig. 5).

SW504 is a *M. xanthus* mutant strain that does not produce cell surface EPS [41]. This mutant is unable to form aggregation centers under developmental conditions (Fig. 5B). Removal of the EPS parameter (Table 1, item 4) from our model reproduces this mutant phenotype, i.e. all cells move independently of each other and do not form big groups (Fig. 5A). Without EPS, the cells are less likely to align with each other and tend to disperse instead of moving side by side. Mechanistically, this is due to the fact that the active turning becomes a dominant factor in determining direction of cell movement as EPS-guided direction no longer plays a role.

SW600 and SW601 are hypo-reversal and hyper-reversal *M. xanthus* mutants, respectively [42]. Different from wild-type cells which reverse every 6–8 minutes and reduce reversal frequency depending on neighboring cell density, SW601 cells constantly reverse approximately every 2 minutes, while SW600 cells only reverse every 120 minutes [16], [42]. The early aggregation phenotypes of these two mutants are measured at 12 hours after induction of development and both are found to form curly aggregate structures consistent with their reported “frizzy” fruiting body phenotypes [16] (Figs. 5D, 5F). By changing the value of the cell reversal frequency parameter (Table 1, item 2) according to the values in mutant strains SW600 and SW601, and turning off the quorum sensing parameter (Table 1, item 3) for reversal modulation in our model, the simulation produces phenotypes similar to the ones observed experimentally for the mutants (Figs. 5C, 5E). Either increasing or decreasing the reversal frequency inhibits the formation of compact early aggregation centers. These agreements suggest that our computational model successfully simulates not only wild-type cell behavior, but also reproduces phenotypes of known genetic mutants during early development of *M. xanthus*.

## Discussion

Understanding how cells interact and the essential elements that facilitate their coordination are important in explaining the social behavior exhibited by many bacterial cells. To achieve this goal, we develop an off-lattice cell-based computational model to simulate gliding behavior and aggregation center formation during *M. xanthus* development. This model is cell-based in which cell movement is determined solely by a motility algorithm and local rules of interaction that are developed based on experimentally determined parameters. Thus, our model offers transparency and flexibility as the advantages in comparison to other off-lattice-based statistical mechanics model.

In this paper, we show that our model successfully reproduces the array of social behaviors of *M. xanthus* at both low and high density, from single cells to the spontaneous formation of multicellular aggregation center involving thousands of cells without the necessity of creation of an artificial traffic jam, as used in some previous models [24], [25]. This automatic formation of aggregation center is supported by the observations of Curtis et al. [43], Holmes et al. [29] and Hendrata et al. [30] in their simulation models. These models included the turning and bending of cells as random fluctuations due to stochasticity in the models' algorithm. The cumulative effect of cell turning and bending enabled the spontaneous aggregation center formation in their simulations. However, our experimental data show that turning angle and frequency are not completely random, but instead follow normal distributions centered at distinct values. The individual cell movement, gliding pattern as well as the timing of aggregation center formation in our simulation closely matches the real cell behaviors observed in recorded experiments.

With this model we test the sufficiency of several previously determined biological elements that are hypothesized to be important for multicellular social behavior during both gliding and early aggregation center formation of *M. xanthus* development. We demonstrate that EPS slime depositing and following, cellular reversals, as well as cell density dependent reversal frequency are essential for these biological processes. In addition, we identify for the first time a new element for social behavior that is necessary for accurately producing the gliding pattern and efficient aggregation center formation - the active turning. Active turning is the tendency of cells to randomly change its direction during movement without the presence of factors such as EPS slime trail or cell collision. This parameter is important in the early stage of development because the cell's tendency to bend its body up to a certain angle determines the initial formation of circular slime trails and determines the EPS slime pattern on surface. The cell turning and curly trajectory of motile cells facilitates the spiral movement during aggregation and helps creating the starting location of dense aggregation centers. Furthermore, we note that the angle for this active turning is very important. Only by turning to a certain preferred angle, 30 degrees in this case, can the cells aggregate most efficiently. While the underlying mechanisms of this active turning behavior are currently unknown, it could be produced by a number of scenarios including random imbalances in motor function or yet to be determined external factors such as nanoscale surface properties. In addition, undetermined myxobacterial cellular features may be involved. Thus, careful examination of mutant strains defective in aggregation while being intact for motility, reversal, EPS production and quorum sensing has the potential to reveal the molecular basis for active turning.

In addition, our model includes the cell density dependent reversal frequency in the simulation. Although this phenomenon is commonly observed, previous models either ignored it or approximated reversal as all-or-none [25], [29], [30], overlooking the importance of cells to quorum sense its environment and adjust their behaviors accordingly. Our model suggests that cell density dependent reversal in *M. xanthus* is indispensable since without it, the efficiency to form aggregation centers decreases. It is known that the Frz chemosensory system is responsible for the reversal events [16]. Methylation or demethylation of FrzCD, the methyl-accepting chemotaxis protein (MCP) of the Frz system may provide bacteria a short term “memory” of concentrations of attractants [15], in this case the EPS slime, to determine the frequency of reversal. Similar to the run-tumble behavior of swimming in E. coli, through controlled reversals, *M. xanthus* may employ a biased random walk [15], [17], [44], [45]. This eventually leads the cells toward places with higher cell concentration and more EPS slime, finally resulting multicellular fruiting body formation. Some previous models [32]–[34] also indicate that slime trails decay over time. As there is no experimental evidence supporting this argument, we omit their decay is our model.

Finally, our model not only provides an approach to decipher the essential minimal factors for individual processes during early aggregation formation, but also proves to be useful to determine how changes in individual behavior due to genetic mutation affect the aggregation formation phenotype (Fig. 5). The model is tested against three known *M. xanthus* mutant strains SW504 (no EPS), SW600 (reduced reversal frequency) and SW601 (elevated reversal frequency) and all three distinct phenotypes are reproduced by changing relevant parameters in the model. Therefore, this computational model can be used as an effective tool to predict mutant social behaviors and phenotypes during development.

Our model provides a tool to understand how interaction between cells facilitates organized movements and which minimal elements are required for these social behaviors. Understanding the cellular social interactions in *M. xanthus* may shed light on the self-organizing processes in fruiting body formation in other Myxobacteria and may also provide insight for the organized movement of cells in the spread of biofilm in an infected tissue and morphogenesis in higher organisms.

## Materials and Methods

### *M. xanthus* development assay

All *M. xanthus* strains used in this study are listed in Table 2. *M. xanthus* cells were grown in CYE medium (1% casitone, 0.5% yeast extract, 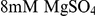 in 10 mM Mops buffer, pH 7.6) at 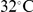 on a rotary shaker at 300 rpm and were maintained on 1.5% CYE agar plates. *M. xanthus* cells were grown in CYE to the exponential growth phase and concentrated to 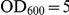 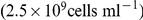 in TPM buffer (10 mM Tris-HCl pH 7.6, 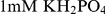, 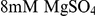). 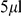 aliquot concentrated cells was spotted onto CF agar [46] and incubated at 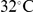. Snapshots were taken every 3 hours during development using a Nikon eclipse TE200 inverted microscope fitted with a SPOT camera (Diagnostic Instruments). Images at 12 hours were used to represent the early aggregation center formation stage.

**Table 2:** *M. xanthus* strains used in this study.

Strain	Relevant genotype	Phenotype	Reference or source
DK1622	Wild-type	Wild-type	[49]
SW504	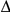 *difA*	No EPS	[41]
SW600	*frzE*::Tn5tet 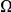 234	Slower reversal	[42]
SW601	*frzD*::Tn5tet 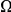 224	Faster reversal	[42]

**Table 3:** Effect of different active turning angle on aggregation center formation.

Ave. turning angle (degrees)	Approx. time needed for aggregation, if possible	Aggregate characteristics
5	More than 20 hours	No round structure formed
15	More than 20 hours	Middle hole persists
30	12 hours	Round and dense, stable
45	9 hours	Round and dense, stable
60	Less than 9 hours	Long aggregates consisting of many small round aggregates that do not merge

### Individual cell motility analysis

Individual cell motility parameters were determined following previously described protocols [47]. Briefly, 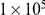 exponentially growing cells (about 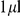) were diluted 10-fold with MOPS buffer and spotted on 1.5% agar MOPS plates. Cell movements were viewed with a Nikon Eclipse TE200 inverted microscope at 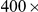 total magnification and recorded with a SPOT camera. Active turning is defined as the ability and tendency of a cell to turn to a certain angle larger than 5 degrees without the presence of any apparent external factors (existing/visible EPS trail or cell collision). The active turning angle was measured by calculating the acute angle between the cell long axis before and after movement of one cell length from an arbitrarily chosen starting point. The active turning intervals were calculated by measuring the amount of time (minutes) per active turning event for a single cell. 200 different cells were examined for each parameter value.

### Detailed description of the computational model

In this subsection, we describe the implementation of each of the experimentally determined parameters (listed in Table 1) into our model.

#### 1. Cell representation and basic properties

**Figure 6:**
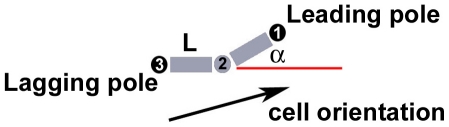
The physical model of *M. xanthus* cell. A cell model with three nodes (N = 3) and its orientation are shown. For algorithm implementation purpose, each cell node is numbered with leading pole as node 1 and lagging pole as node 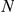. Any two consecutive nodes are connected by a segment of length L. Cell is allowed to turn or bend to some angle 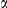.

The computational model for *M. xanthus* we develop here is a two-dimensional off-lattice model in which a cell is represented as a string of N nodes connected by (N-1) segments of equal length L. As the cell length varies between 3–5 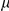
m, the number of nodes N is varied from three to five. Cell width is set to be 0.1 of its length. Three nodes (or two segments) are required minimally for cell bending and turning. Fig. 6 shows a cell with three nodes (N = 3), where the black dots indicate the two poles of the cell. Cell nodes are numbered with the leading pole being node 1. In this representation, cell orientation is defined to be the vector pointing from the lagging pole to the leading pole and it is given by
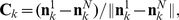
(1)where 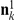 and 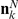 are the positions of leading and lagging poles of cell 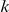, respectively.

In our model we assume that cell movement is determined by the leading pole (node 1). Cell velocity is fixed at 4.5 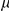
m/min. One simulation time step corresponds to 1/3 minutes. At each iteration, cell 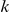 updates its leading pole position according to the discrete equation:
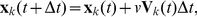
(2)where 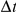 is the time step, 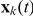 is the position of the leading pole of cell 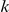 at time 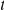, 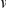 is cell velocity and 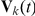 is the motility direction at time 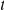. The rest of the nodes get pulled forward following the leading pole, for example, the position of node 2 at time 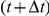 will be the position of node 1 at time 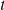, etc. In general, the direction of movement 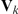 is determined by three possible factors: collision with other cells, EPS-driven direction (depositing and following EPS slime), and the cell's active turning. EPS-driven direction and active turning mechanism will be discussed in item 4 and 5 below. Collision, if it occurs, is primarily resolved according to interaction rules stated in Table 1 item 1. In our simulation, we say the leading pole of cell 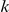 collides with a node of cell 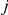 if the distance between them is less than one cell width. There are two cases:

- *Pole-to-side collision* occurs when the leading pole (node 1) of cell 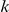 collides with a node of cell 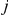 other than the leading pole (node number 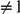). Cell 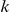 then aligns with cell 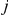. We model the alignment in such a way that cells eventually orient with their neighbors to the acute angle, that is, setting
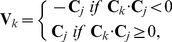
(3)where 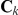 and 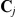 are the orientation of cells 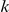 and 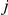, respectively, as defined by (1).
- *Pole-to-pole collision* occurs when the leading pole (node 1) of cell 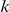 collides with the leading pole (node 1) of cell 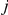. Cell 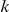 randomly chooses, with uniform probability, to either slightly changes its orientation to pass by the colliding cell and move forward, or reverses its orientation. That is, we have for the current iteration either
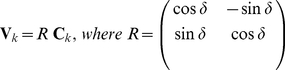
(4)is the rotation matrix with random angle 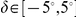, or 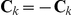.

#### 2. Cellular reversal

Cellular polarity reversal is regulated by a reversal clock that is assigned to each cell. Initially, each cell is assigned a random reversal period 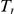 that is normally distributed with mean 6.24 minutes [42]. At the beginning of simulation, each clock is set to a random phase between 0 and 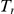. At each iteration, this phase is increased by 1 unit and when it reaches 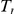, the cell reverses and its clock is reset to 0. During cellular polarity reversal, the cell orientation is reversed (i.e. setting 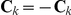), and so are the two cell poles and their functions.

#### 3. Quorum sensing

**Figure 7:**
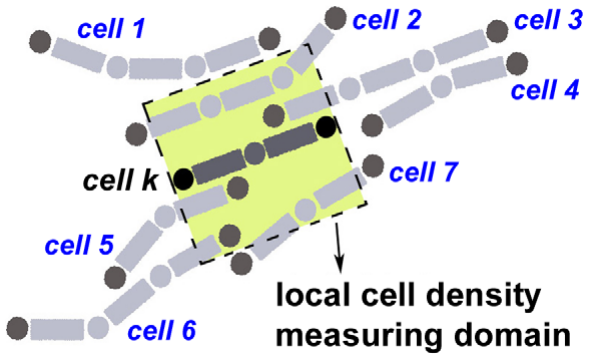
An example illustrating how local cell density is counted. The local measuring domain of a cell is defined to be a square whose sides are taken to be equal to cell length. The measuring domain of cell *k* (colored in dark grey) is the square colored in yellow. Five of the neighboring cells, labeled by number 2, 3, 5, 6 and 7, lie or partly lie within this square, giving the local cell density of cell *k* to be 5. This diagram also illustrates cell turning and alignment of cells in close proximity.

Our model allows the reversal frequency to be modulated during the development according to local cell density. Local cell density is measured by counting the number of neighboring cells that partly or entirely lie within the cell's *measuring domain*, a square region enclosing the cell whose sides equal one cell length. The cell's center of mass is taken to be the center of the measuring domain. Fig. 7 shows a cell, colored in dark grey, having 5 neighboring cells as its local cell density. When a cell reverses, its local density is calculated to see if its current reversal period 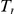 needs to be adjusted according to values in Table 1 parameter 3 - quorum sensing.

#### 4. EPS production and EPS-driven direction

In all myxobacterial cells, except the EPS- mutants, the EPS-driven direction becomes more prominent than direction due to active turning as cells tend to follow slime trails previously laid on the surface. In our algorithm, we take into consideration both the effect of exopolysaccharide (EPS) slime secretion from the rear pole and the cells' tendency to follow the slime trail deposited previously by other cells.

**Figure 8:**
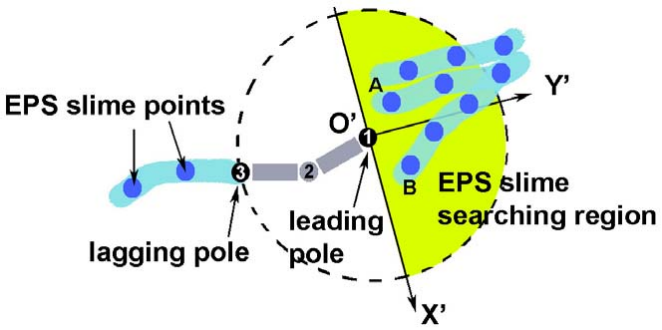
A model for EPS-driven cell movement. A cell deposits EPS slime from its lagging pole (node 3) as it moves while searching for existing EPS slime in the vicinity of its leading pole (node 1).

Our model is essentially a discrete model in which the cell position is stored by keeping track of the position of each cell node in a two-dimensional domain. Similarly, we keep track of the EPS slime trail by keeping track of the position of the EPS points deposited on the surface from cell's lagging pole. EPS slime secretion pushes the cell directly forward, causing the cell to orient itself along its long axis given by (1). In Fig. 8, a cell leaves behind a light blue trail representing the slime with EPS points in it.

In addition to depositing EPS, a cell also has the capability to sense and follow a slime trail previously deposited by other cells. Slime reduces surface friction and thus moving on slime trail requires less energy than moving on a dry surface. This reflects in the tendency of a cell to turn to an acute angle voluntarily whenever it encounters a slime trail. To model this behavior, we first define *EPS slime searching region* to be the semi-circular region right ahead of the leading pole with radius approximately one cell length. This EPS searching region indicates how far a cell can sense EPS trail nearby and indicates possible region to which a cell can move towards. A cell searches for EPS points inside this searching region and turn to an acute angle to follow the trail on which these EPS points are located. If there are more than one slime points within the EPS searching area that satisfy the acute angle requirement, then the cell moves towards the region with more EPS slime points. In experiments, this corresponds to the region with higher EPS concentration. Fig. 8 illustrates a cell with a yellow semi-circular region indicating its EPS searching area. Here, the cell turns in the direction towards the slime point 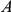 since the sub-region in which 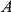 lies has 5 slime points, while the sub-region in which slime point 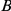 is located only has 2. Thus, the direction due to slime orientation is given by:
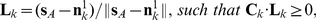
(5)where 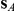 is the position of the slime point A. The inequality in (5) ensures that cell turns to an acute angle in following a slime trail.

#### 5. Active turning mechanism

The active turning mechanism relies on two parameters, namely its frequency and turning angle. Each cell is assigned two variables; one to keep track of the time of the last active turning and the other one is a random active turning interval 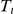 whose value follows a normal distribution around 3 minutes per turn. When a moving cell neither collides with another cell nor encounters a slime trail, its active turning takes place provided that the time interval since the last active turning is equal to or has exceeded 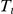. Otherwise, the cell moves following its tail-to-head orientation defined by equation (1).

Thus, the direction of movement 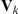 of cell 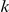 in equation (2) then becomes
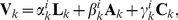
(6)where 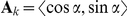 and 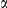 is the active turning angle. The weights 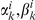 and 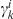 at each iteration 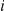 is determined based on the following motility algorithm:

- Checking the reversal clock and quorum sensing. If the phase of the cell's reversal clock has reached its reversal period 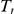, cell reverses and we set the weights 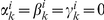 as cell does not move at this particular iteration. Local cell density is then computed to check whether the current reversal period 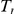 needs to be modulated. If the cell does not reverse, go to step (b).
- Checking for collision with other cells. If there is collision, resolve it according to collision algorithm (equation (3) or (4)), and set 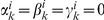. If there is no collision, proceed to step (c).
- Searching for slime trail within the EPS slime searching area. If an EPS slime is found, compute equation (5) and set 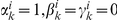. Otherwise, proceed to step (d).
- Checking for active turning time interval. If the time interval since the last active turning is equal to or greater than 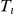, then 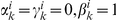. Otherwise, cell moves in the direction of its tail-to-head orientation (1) and we set 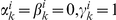.
